# Conservative management of grade 1V renal injury with complete transection: a case report

**DOI:** 10.1186/1757-1626-1-129

**Published:** 2008-08-27

**Authors:** Costa Healy, Mohamed Hobeldin, Anies Mahomed

**Affiliations:** 1 Department of Paediatric Surgery, Royal Alexandra Children's Hospital, Brighton, UK; 2 Department of Paediatric Surgery, Sheikh Khalifa Medical Centre, Abu Dhabi, United Arab Emirates

## Abstract

The expectant management of high grade renal injuries in hemodynamically stable children has gained increasing acceptance amongst paediatric surgeons. However, patients with grade 1V injury with complete renal transection have been identified as a subgroup with a poor outcome that may benefit from early operative intervention.

Interestingly, both internal and external drainage have been independently utilised as part of the expectant approach. The former is more widely practiced and was first suggested by Haas et al who used it successfully in 5 patients with grade 1V renal trauma. Yet to be clearly established in this context is the value and timing of external drainage, particularly, when used in combination with internal stenting.

Described is a child with complete renal transaction who was successfully managed with a combination of internal and external drainage.

## Case presentation

**Figure 1:**
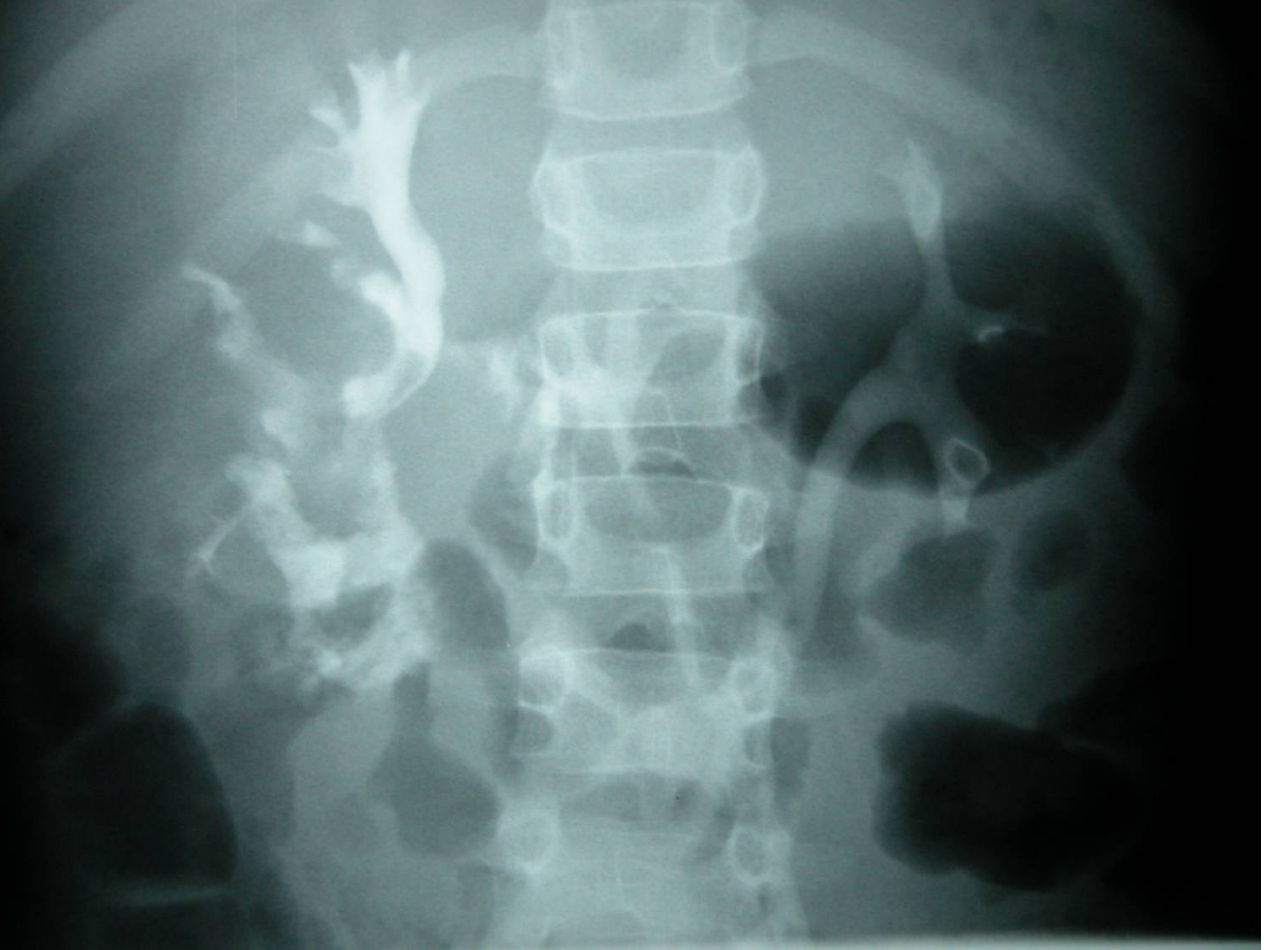
Polar diastasis and urinary extravasation as seen on IVP.

A 5 year old Caucasian male patient was admitted to a tertiary paediatric surgical centre within 1 hour of coming off a quadbike, sustaining blunt trauma to the right chest and the right upper quadrant of abdomen. Initial assessment confirmed hypovolaemic shock which responded to aggressive resuscitation as well as a tender right flank with macroscopic haematuria. Once stabilised a Computarised Tomography (CT) scan and IVP were performed which confirmed contusion of the right lower lung, fracture of several overlying ribs and complete right renal fracture with distraction of upper and lower poles (Grade1V), (Figure 1). Also documented were contrast extravasation from the pelvico-calyceal system and a substantial perinephric haematoma. Significantly, some contrast was noted in the distal right ureter confirming ipsilateral pelvico-ureteric continuity.

The patient was subsequently admitted to the intensive care unit where he was managed with bed rest, broad spectrum antibiotic cover and regular haemoglobin checks with transfusion top ups as necessary.

Over the next day the patient developed an ileus with progressive abdominal distended and an ultrasound scan was performed. This confirmed an expanding right perinephric collection which was managed by sonar guided placement of a percutaneous pigtail catheter – maintained on free drainage.

**Figure 2:**
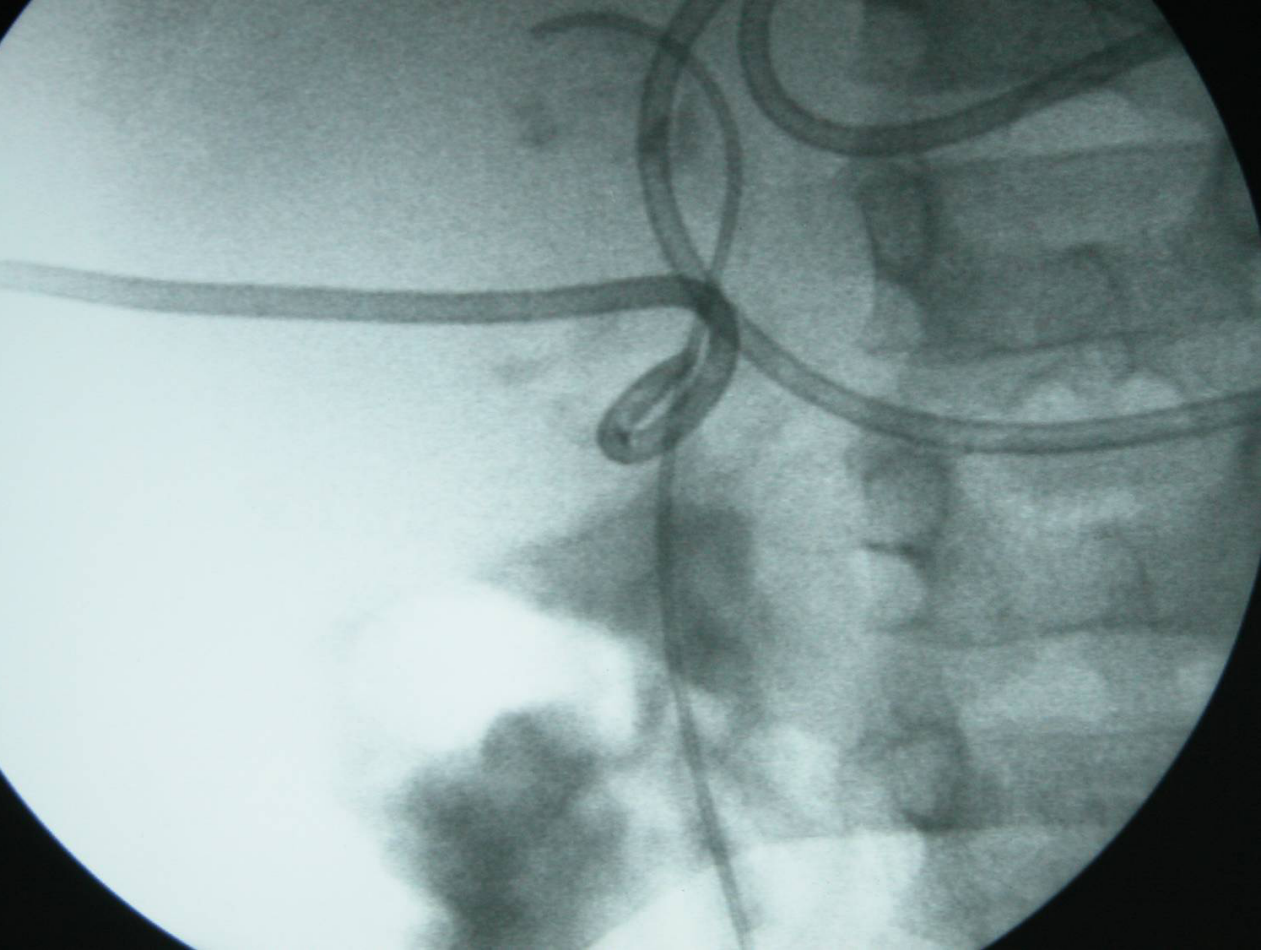
Concomitant percutaneous pigtal and internal double J drainage of right kidney.

Twenty four hours later with worsening abdominal distension and high output drainage from the pigtail catheter, the child was taken to theatre where under a general anaesthetic and fluoroscopic guidance a 4.5 French double J stent was passed retrogradely into the right renal pelvis. (Fig 2) This had the immediate effect of diverting urine from the pigtail catheter whose output dropped significantly. Macroscopic haematuria and the ileus improved gradually over the next 10 days allowing for the resumption of oral feeding and withdrawal of total parental nutrition.

The pigtail catheter was removed on cessation of urine drainage at 3 weeks. A duplex scan at this point demonstrated resolution of the perinephric collection with greater approximation of the renal poles. A repeat ultrasound scan at 5 weeks with the double J stent still in situ showed radiological evidence of a reconstituted kidney. The patient was discharged at this stage and readmitted 3 weeks later for removal of the ureteric stent.

**Figure 3:**
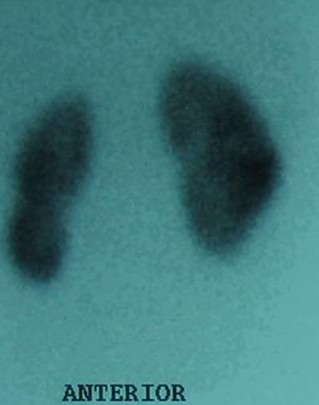
DMSA Scan demonstrating a slightly elongated but otherwise normally functioning right kidney.

His subsequent recovery was uneventful and at 4 years post injury he remains normotensive with equitable renal function noted on Dimecaptosuccinic acid (DMSA) scan (Fig 3)

## Discussion

The kidneys in children are proportionally larger and have much less perirenal fat and costal protection than in adults which predisposes them to injury after blunt abdominal trauma (10% of cases) [1,2]. Prompt attention to these injuries is vital to allow for optimal recovery and to minimise long term sequelae. The management of renal trauma ranges from an emergency laparotomy for haemodynamic compromise to observation without intervention in minor lacerations. As with all solid organ injuries in children there has been a shift to conservative management where possible [1,2]. Increasingly minimally invasive techniques are being adopted to manage significant renal trauma where there is no haemodynamic compromise [2-5]. This approach results in a lower incidence of nephrectomy and has few long term complications.

However the management of grade IV renal injury in children is controversial, particularly with regard to the management of urinary extravasation. Grade IV trauma is defined as parenchymal laceration extending through the corticomedullary junction and into the collecting system [6]. This results in urinary extravasation with the potential for large urinomas. Traditionally these injuries have been treated aggressively with open surgery with the justification that urinomas may lead to perirenal fibrosis with complications of obstruction, infection and hypertension. However much of this evidence is from the adult population and is not necessarily applicable to paediatric practice [6]. The options for treating urinomas are; open drainage, with or without surgical repair, ureteric stents, percutaneous drains or just observation.

Grade 1V renal injuries with complete fracture and separation of the poles but with intact blood supply constitutes a special group with a decreased likelihood of spontaneous resolution and where early intervention may be necessary [1]. Endourological stenting or percutaneous drainage and failing this, open surgery, are options described in the management these patients. Endourological stenting on its own has not gained widespread popularity because stent calibre is not thought to be sufficient for optimal drainage [1].

There is currently no consensus on the optimal timing of these interventions. However from the experience of this case where a policy of early aggressive management with a combination of internal and external drainage was successfully utilised would lead us to support this approach to improve renal salvage. It would appear that reconstitution of the distracted but viable renal poles into a solitary functioning unit is possible provided the urinoma is adequately managed. Successful drainage also ensured that there was no significant residual scarring on late DMSA scanning.

The expectant management of isolated high grade renal injuries with complete fracture in the haemodynamically stable patient is evolving and both internal and percutaneous drainage are crucial to the success of this approach. It seems the advances in interventional radiology are facilitating a less invasive approach to the management of these significant injuries in the paediatric population. The experience of this case reinforces a strategy which is leading to kidney salvage with minimal complications and is advocated.

## Consent

Consent was obtained from both parents and the involved institutions for publication of the case report.

## Competing interests

The authors declare that they have no competing interests.

## Authors' contributions

CH prepared the manuscript, MH provided the clinical details of the case, AM managed the case and edited the paper.

## References

[B1] Rogers CG, Knight V, Macura KJ, Ziegfeld S, Paidas CN, Matthews RI (2004). High Grade Renal Injuries in Children- Is Conservative Management Possible. Urology.

[B2] Russell RS, Gomelsky A, McMahon DR, Andrews D, Nasrallah PF (2001). Management of Grade IV Renal Injury in Children. J Urol.

[B3] Henderson CG, Sedberry-Ross S, Pickard R, Bulas DI, Duffy BJ, Tsung D, Eichelberger MR, Belman AB, Rushton HG (2007). Management of High Grade Renal Trauma: 20-Year Experience at a Pediatric Level I Trauma Center. J Urol.

[B4] Haas CA, Reigle MD, Selzman AA, Elder JS, Spirnak JP (1998). Use of Ureteral Stents in the management of major renal trauma with urinary Extravasation: Is there a role?. J Endourol.

[B5] Buckley JC, McAninch JW (2006). Selective management of isolated and nonisolated grade 1V renal injuries. J Urol.

[B6] Margenthaler JA, Weber TR, Keller MS (2002). Blunt Renal Trauma in Children: Experience with Conservative Management at a Pediatric Trauma Center. J Trauma.

